# Histone deacetylases: the critical enzymes for microglial activation involved in neuropathic pain

**DOI:** 10.3389/fphar.2025.1515787

**Published:** 2025-03-06

**Authors:** Yi Ding

**Affiliations:** Department of Anesthesiology, The Affiliated People’s Hospital of Ningbo University, Ningbo, China

**Keywords:** HDACs, microglial activation, neuropathic pain, mechanism, inhibitor, activator

## Abstract

Neuropathic pain is a common health problem in clinical practice that can be caused by many different factors, including infection, ischemia, trauma, diabetes mellitus, nerve compression, autoimmune disorders, cancer, trigeminal neuralgia, and abuse of certain drugs. This type of pain can persistently affect patients for a long time, even after the rehabilitation of their damaged tissues. Researchers have identified the crucial role of microglial activation in the pathogenesis of neuropathic pain. Furthermore, emerging evidence has shown that the expression and/or activities of different histone deacetylases (HDACs) can modulate microglial function and neuropathic pain. In this review, we will summarize and discuss the functions and mechanisms of HDACs in microglial activation and neuropathic pain development. Additionally, we will also list the emerging HDAC inhibitors or activators that may contribute to therapeutic advancement in alleviating neuropathic pain.

## 1 Introduction

The definition of neuropathic pain has been developed over the past 30 years. In 1994, International Association for the Study of Pain (IASP) described that neuropathic pain as “pain initiated or caused by a primary lesion or dysfunction in the nervous system”. Subsequently, to further clarify and develop the concept of neuropathic pain, some experts defined it as “pain arising as a direct consequence of a lesion or disease affecting the somatosensory system”, a definition that has been endorsed by IASP. Studies have estimated that nearly 1 in 10 people have experienced neuropathic pain (Scholz et al., 2019; Cavalli et al., 2019). A detailed description of the incidence and prevalence of neuropathic pain has been conducted by Kerstman et al. (2013). Common causes of neuropathic pain include infection, ischemia, trauma, diabetes mellitus, nerve compression, autoimmune disorders, cancer, trigeminal neuralgia, and abuse of certain drugs (Basic-Kes et al., 2009; Fornasari, 2012). Neuropathic pain is classified based on its site of occurrence into two main categories: peripheral and central neuropathic pain. Peripheral neuropathic pain includes diabetic neuropathy, trigeminal and post-herpetic neuralgia, persistent post-operative and post-traumatic pain, complex regional pain syndrome, cancer-related neuropathic pain, HIV-related neuropathic pain, and pain after amputation. Central neuropathic pain encompasses post-stroke pain, pain after spinal cord injury (SCI), central pain in Parkinson’s disease or other neurodegenerative diseases, pain associated with syringomyelia and multiple sclerosis (Szczudlik et al., 2014). Although the manifestations of neuropathic pain varies among patients, common diagnostic features include allodynia (pain in response to non-noxious stimuli), hyperalgesia (increased sensitivity to pain), and spontaneous pain (Jensen and Finnerup, 2014; Bennett, 2012). Unfortunately, neuropathic pain can continuously affect patients for a long time, even after the rehabilitation of their damaged tissues. Therefore, researchers have made efforts to elucidate the pathogenesis of neuropathic pain and find ways to alleviate it.

Different animal models of neuropathic pain have been developed to study the mechanisms and effective treatment strategies for this condition. Numerous traumatic nerve injury models exist, like spinal nerve ligation (SNL), partial sciatic nerve ligation (pSNL), chronic constriction injury (CCI) to the sciatic nerve, brachial plexus avulsion (BPA), sciatic nerve transaction (SNT), and sciatic nerve trisection (Challa, 2015; Jaggi et al., 2011). Furthermore, pain models based on chemotherapeutic agents, HIV, ethanol, diabetes, cancer, and others have also been employed to understand the pathogenesis and manage pain due to their respective etiologies in clinical settings [reviewed in Jaggi et al. (2011), Casaril et al. (2024)].

Microglia are specialized immune cells that reside within the central nervous system (CNS), which includes the brain and spinal cord. They constitute the first line of immune defense and play crucial roles in maintaining homeostasis and immune surveillance within the brain and spinal cord (Kettenmann et al., 2013). Microglia-induced chronic inflammation is a vital pathological factor in neuropathic pain. To better understand the pathogenesis of this condition, researchers commonly use lipopolysaccharide (LPS)-stimulated microglia as an *in vitro* model (Li X. et al., 2023; Hua et al., 2024). Histone deacetylases (HDACs) are a class of enzymes that remove acetyl groups from histones and non-histone proteins, thereby regulating gene expression and other cellular processes (Kulthinee et al., 2022). Emerging evidence has highlighted the crucial role of microglial activation in the pathogenesis of neuropathic pain (Tozaki-Saitoh and Tsuda, 2019; Tsuda, 2019). Moreover, the activities of different HDACs participate in the regulation of microglial function and neuropathic pain (Lin et al., 2015; Nakamura et al., 2020; Liao et al., 2020). Hence, in this review, we will summarize and discuss the functions and mechanisms of HDACs in microglial activation and neuropathic pain development. Additionally, we will list emerging HDAC inhibitors or activators that may contribute to therapeutic advancements in alleviating neuropathic pain (Tables 1, 2).

**TABLE 1:** Effects of HDAC inhibitors in microglial activation and neuropathic pain.

Inhibitor	HDAC specificity	Model/Disease	Mechanism of action	Results	Ref
CI-994	Class I	SCI mouse model (*in vivo*)	Enhanced acetylated histone H3 expression in microglia/macrophages	Promoted behavioral recovery	Zhang et al. (2018)
Zingiberene	HDAC1	Spared nerve injury mouse model (*in vivo*)	Downregulated HDAC1 levels in the spinal cord microglia	Reduced thermal hyperalgesia and mechanical allodynia	Borgonetti et al. (2023)
Scriptaid	Class I/II	Intracerebral hemorrhage mouse model (*in vivo*)	Shifted microglia/macrophage polarization toward the M2 phenotype, reduced proinflammatory cytokine secretion	Improved neurological functional recovery	Yang et al. (2021)
Scriptaid	Class I/II	A transwell co-culture model of primary microglia and oligodendrocytes (*in vitro*)	Modulated microglia polarization towards M2 phenotype and mitigated neuroinflammation	Protected oligodendrocytes	Yang et al. (2021)
Apicidin, MS-275, and MI-192	Class I	LPS-induced injury in microglia BV2 cells (*in vitro*)	Suppressed the expression of IL-6 and TNF-α	Inhibition of microglial activation	Durham et al. (2017)
CAY10683	HDAC2	LPS-activated BV2 microglial cells (in vitro) and LPS induced mice (*in vivo*)	Inhibited expression levels of TNF-α and IL-1β in both models by decreasing HDAC2/TLR4/NF-κB signaling pathway	Mitigated neuroinflammation	Jiao et al. (2018)
RGFP966	HDAC3	SCI mouse model (*in vivo*)	Inhibited microglial activation and inflammatory response via Sirt1/Nrf2 signaling pathway	Improved neurological function recover	Chen et al. (2023a)
Valproic acid	Class I	SCI mouse model (*in vivo*)	Promoted the phenotypic switch of microglia M1 to M2 and inactivated microglia to reduce inflammatory through STAT1-mediated acetylation of the NF-κB pathway, dependent of HDAC3 activity	Improved locomotion recovery	Chen et al. (2018a)
Valproic acid	Class I	SNL rat model (*in vivo*)	Downregulated proinflammatory cytokines and HDAC3 expression, upregulated anti-inflammatory cytokines, inhibited spinal microgliosis and promoted microglia polarization to the M2 phenotype via the STAT1/NF-κB and JAK2/STAT3 signaling pathways	Alleviated mechanical allodynia	Guo et al. (2021)
BRD3308	HDAC3	Intraventricular hemorrhage mouse model (*in vivo*)	Decreased neuronal loss, microglial activation, and pyroptosis in the hippocampus partly by activating the PPARγ/NLRP3/GSDMD signaling pathway	Improved neurobehavioral performance	Li et al. (2023b)
BG45	Class I with selectivity of HDAC3	Alzheimer’s disease mouse model (*in vivo*)	Decreased Aβ deposition, downregulated phosphorylation of tau protein and the expression of inflammatory cytokines, reduced IBA1-positive microglia and GFAP-positive astrocytes	Reduced the number of degenerative neurons and the activation of microglia and astrocytes	Wang et al. (2023)
ACY-1215	HDAC6	CCI mouse model (*in vivo*)	Inhibited neuron activation and suppressed pyroptosis and neuroinflammatory responses in spinal cells	Alleviated mechanical allodynia	Sun et al. (2023)
PB131	HDAC6	LPS challenged microglia BV2 cells and model mice *(in vitro* and *in vivo*)	Displayed good brain permeability, high specificity, and strong potency toward inhibiting HDAC6	Suppressed neuroinflammation	Liu et al. (2023)
Compound 5	HDAC11	LPS-induced microglia and depression mouse model (*in vitro* and *in vivo*)	Initiation of autophagy and inhibition of nitric oxide *in vitro* and inhibited microglial activation in mouse brain	Attenuated microglial activation in both models, alleviated depression-like behavior *in vivo*	Baek et al. (2023)

LPS, lipopolysaccharide; SCI, spinal cord injury; SNL, spinal nerve ligation; CCI, chronic constriction injury.

**TABLE 2:** Effects of HDAC activators in microglial activation and neuropathic pain.

Activator	HDAC specificity	Model/Disease	Mechanism of action	Results	Ref
SRT2104	Sirt1	OGD/R-injured neurons and microglia (*in vitro*)	Shifted microglia to M2 phenotype, inhibited the activation of NF-κB and enhanced Sirt1 expression in microglia	Alleviated OGD/R induced microglial death and neuronal death	Fu et al. (2021)
SRT1720	Sirt1	Subarachnoid hemorrhage rat model (*in vivo*) neurons and microglia co-culture system (*in vitro*)	Enhanced Sirt1 expression, promoted microglial M2 polarization, diminished oxidative stress and NLRP3 inflammasome activation	Improved neurological functions and ameliorated neuronal death i*n vivo*; alleviated neuronal damage *in vitro*	Xia et al. (2021)
2,3,5,6-Tetramethylpyrazine	Sirt1	LPS-induced BV2 cells and mouse models (*in vitro* and *in vivo*)	Reduced the levels of proinflammatory cytokines and chemokines, promoted microglial M2 polarization	Alleviated neuroinflammation	Chen et al. (2023b)
Omega-3 polyunsaturated fatty acids	Sirt1	TBI rat model (*in vivo*)	Promoted M2 microglial polarization, inhibited microglial activation, downregulated HMGB1 acetylation and its extracellular secretion	Attenuated neuroinflammation	Chen et al. (2018b)
A3	Sirt1	TBI rat model (*in vivo*)	Suppressed microglial activation and proinflammatory factor expression, inhibited PGC-1α and Nrf2 nuclear translocation and secretion	Inhibited neuroinflammation	Chen et al. (2022)
Pterostilbene	Sirt1	Subarachnoid hemorrhage rat model (*in vivo*)	Attenuated microglia activation, oxidative insults, and neuronal damage, enhanced Sirt1 expression, and promoted Nrf2 accumulation in nuclei	Protected brain from injury	Zhang et al. (2022)
Cycloastragenol	Sirt1	Middle cerebral artery occlusion mouse model (*in vivo*)	Reduced brain infarct volume, ameliorated functional deficits, prevented neuronal cell loss, inhibited the activation of microglia and astrocytes	Protected against ischemic brain injury	Li et al. (2020b)
P7C3-A20	Sirt3	Intracerebral hemorrhage mouse model (*in vivo*)	Diminished lesion volume, reduced blood-brain barrier damage, mitigated brain edema, attenuated neural apoptosis and microglial activation	Improved neurological outcomes, suppressed neuroinflammation	Wu et al. (2023)

OGD/R, oxygen glucose deprivation/reoxygenation; LPS, lipopolysaccharide; TBI, traumatic brain injury.

## 2 Microglial activation in the pathogenesis of neuropathic pain

Microglia play crucial roles in maintaining homeostasis and immune surveillance within the CNS (Zhu et al., 2023). Although the exact origin of microglia is still debatable, it is widely accepted that microglia originate from primitive macrophages in the yolk sac during early embryonic development. These progenitor cells populate the brain and spinal cord and then differentiate into microglia in normal conditions throughout the development (Ponomarev et al., 2013). Moreover, bone marrow-derived myeloid cells also migrate into the CNS and differentiate into microglia under pathological conditions such as neuroinflammation (Ponomarev et al., 2013). Microglia have a highly ramified morphology with numerous thin, branched processes that extend throughout the neuropil. This morphology allows them to efficiently survey their microenvironment and interact with neurons, synapses, and other glial cells (Cornell et al., 2022; Wu and Eisel, 2023). Microglia continuously monitor the brain for signs of injury, infection, or disease (Stonedahl et al., 2020). They are capable of detecting and responding to various stimuli, such as pathogens, damaged neurons, and abnormal protein deposits. In response to these stimuli, microglia can express and secrete neuroprotective factors that promote neuronal survival and recovery (Bellver-Landete et al., 2019; Carroll and Chesebro, 2019; Jo et al., 2022). Indeed, a study has found that following peripheral nerve injury-induced pain hypersensitivity, CD11c^+^ spinal microglia emerge and facilitate functional recovery by expressing insulin-like growth factor 1 (Kohno et al., 2022). These findings suggest a beneficial role of a small population of microglia in pain recovery.

The correlation between microglial activation and neuropathic pain has been extensively investigated. Upon activation, microglia undergo morphological changes, becoming more ameboid and migrating to the site of injury or inflammation. This activation process is accompanied by the release of a range of mediators, including proinflammatory cytokines, chemokines, and neurotrophic factors (Tozaki-Saitoh and Tsuda, 2019; Tsuda, 2019). Activated microglia exhibit two polarization types: the classically activated microglia (M1 type, proinflammatory) and the alternatively activated microglia (M2 type, anti-inflammatory) (Karavis et al., 2023). Through the release of proinflammatory cytokines and chemokines, activated M1 microglia can sensitize nociceptors (pain-sensing neurons) and enhance their responsiveness to noxious stimuli. This sensitization process contributes to hyperalgesia and allodynia observed in neuropathic pain conditions (Inoue and Tsuda, 2018). Additionally, activated M1 microglia can influence pain perception and modulation through their interactions with the immune system. They can recruit and activate other immune cells, such as macrophages and T cells, further amplifying the inflammatory response and contributing to the chronicity of neuropathic pain (Tsuda, 2019; Zhao et al., 2017). Conversely, when microglia exhibit M2 phenotypes, they secrete anti-inflammatory cytokines and nutrient factors to inhibit inflammation, promote repair and regeneration, and restore homeostasis (Atta et al., 2023; Tu et al., 2021). Studies have confirmed that M2 phenotype microglia can reduce neuropathic pain (Atta et al., 2023; Celik et al., 2020). Interestingly, a recent study has found that although the application of nucleus pulposus (NP) of the intervertebral disc to the sciatic nerve induced a series of neuropathic pain behaviors, it did not cause microglial activation (Tu et al., 2022). Meanwhile, microglial inhibition did not attenuate pain hypersensitivity (Tu et al., 2022). They also found that the cells within the NP could recruit macrophages to the adjacent nerve, which was responsible for pain hypersensitivity (Tu et al., 2022). The intricate roles of microglia in neuropathic pain necessitate a comprehensive elucidation of the mechanisms by which their activation and function are modulated.

## 3 HDACs and their potential role in microglial activation

### 3.1 HDACs: function and isoforms

HDACs are a class of enzymes that remove acetyl groups from histones and other proteins, regulating gene expression and other cellular processes. The primary function of HDACs is to remove acetyl groups from lysine residues of histones, leading to a decrease in histone acetylation. This deacetylation process results in a more compact chromatin structure that is less accessible to transcription factors, thereby repressing gene expression. Additionally, HDACs regulate the acetylation status of non-histone proteins, including transcription factors, cytoskeletal proteins, and metabolic enzymes, further affecting various cellular processes (Luan et al., 2022; Zhang L. Y. et al., 2024; Luo et al., 2019).

Mammalian cells express multiple isoforms of HDACs. In humans, there are 18 known HDAC genes that encode different isoforms. The HDAC family can be further classified into four classes based on their sequence similarity, subcellular localization, and action manners. Class I HDACs include HDAC1, HDAC2, HDAC3, and HDAC8, which are primarily located in the nucleus. Class II HDACs are further subdivided into IIa (HDAC4, HDAC5, HDAC7, and HDAC9) and IIb (HDAC6 and HDAC10), which are found both in the nucleus and cytoplasm. Class III HDACs, also known as sirtuins, include Sirt1 to Sirt7, are located in different subcellular compartments. Class IV HDACs are represented by a single enzyme, HDAC11, which is found in the nucleus. Classes I, II, and IV are zinc-dependent HDACs, while Class III HDACs are NAD^+^-dependent enzymes (Luan et al., 2022; Zhang L. Y. et al., 2024).

### 3.2 HDACs in microglial activation and neuropathic pain: epigenetic regulation and signaling pathways

#### 3.2.1 HDAC1 and HDAC2

**FIGURE 1:**
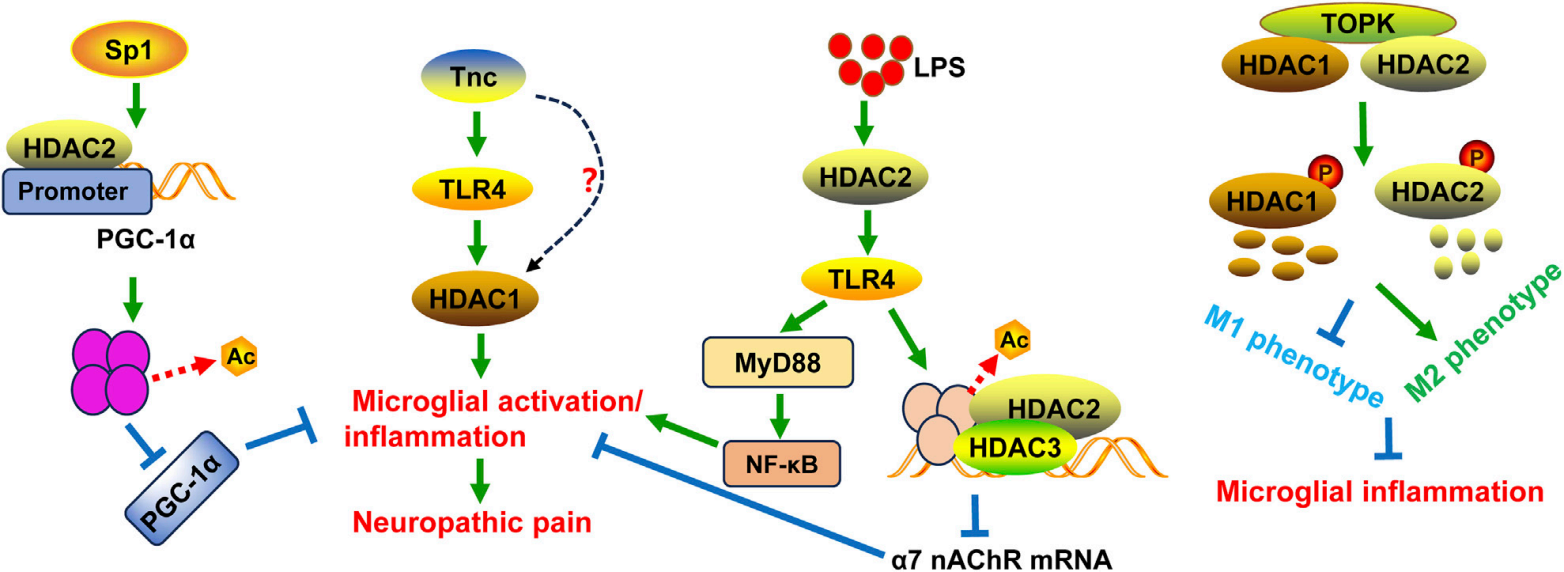
Schematic diagram of the potential mechanisms of HDAC1 and HDAC2 in microglial activation and/or neuropathic pain. Tnc can stimulate HDAC1 expression and activity in microglia, at least partly in a TLR4-dependent manner. Specificity protein 1 (Sp1) seduces histone deacetylation in the PGC-1α promoter and inhibits PGC-1α expression with the help of HDAC2, leading to neuropathic pain and inflammation as well as mitochondrial dysfunction. T-LAK-cell-originated protein kinase (TOPK) can bind to HDAC1 and HDAC2 and regulate their phosphorylation and degradation, thereby shifting microglia/macrophage polarization toward the M2 phenotype. This action can inhibit microglial inflammation. LPS can induce neuroinflammation through the HDAC2/TLR4/MyD88/NF-κB pathway. Additionally, TLR4 activation by LPS downregulates the expression of α7 nAChR mRNA through histone H3 deacetylation mediated by HDAC2 and HDAC3.

HDAC1 and HDAC2 are indispensable for microglial development in embryo, as their absence activates pro-apoptotic genes, increases microglial apoptosis, and reduces survival (Datta et al., 2018). In contrast, HDAC1 and HDAC2 are not necessary for microglial survival in adult (Datta et al., 2018). Tenascin C (Tnc) is an extracellular matrix glycoprotein expressed during embryo development (Giblin and Midwood, 2015). Microglia express a diverse array of pattern recognition receptors (PRRs), including toll-like receptors (TLRs). TLRs can recognize both endogenous and exogenous ligands, thereby orchestrating innate and adaptive immune responses to infection, inflammation, and tissue injury (Haage et al., 2019). Tnc regulated microglial phagocytic activity during early postnatal CNS development partially dependent on microglial TLR4 expression (Haage et al., 2019). Additionally, Tnc’s role during early postnatal development also involved positively modulating HDAC1 levels, a process that was not mediated by TLR4 signaling. The authors also discovered that Tnc induced proinflammatory cytokine/chemokine production, chemotaxis, and phagocytosis in primary microglia via regulating TLR4 signaling and HDAC1 activity. Although 1 µM MS-275 acts on HDAC1, HDAC2, and HDAC3, 300 nM MS-275 specifically inhibits HDAC1. HDAC1 inhibition by 300 nM MS-275 decreased Tnc-induced microglial production of IL-6 and TNF-α. Moreover, the regulation of Tnc on HDAC1 activity was partly in a TLR4-dependent fashion (Haage et al., 2019). Interestingly, the expression of HDAC1 correlated with senescence markers in mouse and human hippocampal biopsies, suggesting its role during physiological aging (Auzmendi-Iriarte et al., 2022). The involvement of HDAC1 and HDAC2 in microglial activation and neuropathic pain following nerve injury has been demonstrated in many studies. Nerve injury induced by pSNL in a mouse model results in mechanical allodynia and heat hyperalgesia, accompanied by increased HDAC1 expression in microglia in the ipsilateral superficial dorsal horn (Kami et al., 2016). However, running exercise can attenuate neuropathic pain and decrease HDAC1 expression (Kami et al., 2016). Direct inhibition of class I HDAC using CI-994 enhances acetylated histone H3 expression in microglia/macrophages in the spinal cord following injury and promotes behavioral recovery (Zhang et al., 2018). Zingiberene (ZNG) has been proposed to suppress HDAC1 activity through a non-zinc-binding mechanism. ZNG treatment can reduce thermal hyperalgesia and mechanical allodynia in animals with neuropathy and downregulate HDAC1 levels in the spinal cord microglia (Borgonetti et al., 2023). HDAC2 expression in CD16/CD32^+^ proinflammatory microglia could be induced by traumatic brain injury (TBI). Unexpectedly, the elevation of proinflammatory cytokines/chemokines and brain infiltration of neutrophils and B cells were observed in microglia-specific HDAC2 knockout mice, suggesting HDAC2 was dispensable for neuroinflammation after TBI (Zhang Y. et al., 2024). In contrast, conditional knockout of the *Hdac2* gene in microglia and HDAC inhibition with scriptaid both improved neurological functional recovery, reduced white matter injury (WMI), and decreased proinflammatory cytokine secretion after intracerebral hemorrhage (ICH) (Yang et al., 2021). The distinct role of HDAC2 in microglia might be related to different animal models. Specificity protein 1 (Sp1) is a member of a family of transcription factors and is implicated in the regulation of many cellular genes that contain putative CG-rich Sp-binding sites in their promoters. CCI upregulates Sp1 expression, which seduces histone deacetylation in the PGC-1α promoter and inhibits PGC-1α expression with the help of HDAC2. Both Sp1 knockdown and PGC-1α overexpression attenuates CCI-induced neuropathic pain, microglia activation, inflammatory responses in spinal cord tissues. Also, silencing Sp1 and PGC-1α upregulation diminishes LPS-induced microglial inflammation and neuronal dysfunction *in vitro* (Miao et al., 2022) (Figure 1).

Mechanically, HDAC1 and HDAC2 expression is tightly correlated with the phenotypic transformation of microglia. In BV2 microglia, suppressing HDAC1 expression can promote the M2 phenotypic transformation of microglia, while overexpression of HDAC1 can mitigate this transformation (Wang et al., 2017). T-LAK-cell-originated protein kinase (TOPK) is a novel member of the mitogen-activated protein kinase (MAPK) family. TOPK can bind to HDAC1 and HDAC2, regulate their phosphorylation, and thereby induce their degradation (Han et al., 2018). Subsequently, TOPK prevents the M1 transformation through reducing the release of inflammatory factors associated with the M1 phenotype and increasing the production of cytokines of M2 phenotype (Han et al., 2018). Moreover, TOPK regulates the M2 phenotype *in vivo* through modulation of HDAC1 and HDAC2 activity (Han et al., 2018) (Figure 1). HDAC inhibition using scriptaid not only shifts microglia/macrophage polarization toward the M2 phenotype after ICH *in vivo*, but also protects oligodendrocytes by modulating microglia polarization and mitigating neuroinflammation in a transwell co-culture cell model (Yang et al., 2021).

In addition, HDAC1 and HDAC2 are critical for microglial activation and inflammation following infection. A previous study has found that following microglial activation, HDAC2 was enriched in gene promoters and mainly regulated gene expression involved in immune- and inflammation-related pathways, such as nitric oxide synthase (NOS) biosynthetic process, retinoic acid-inducible gene- (RIG-) like receptor signaling pathway, and nuclear factor kappa B (NF-κB) signaling pathway (Guo et al., 2020). Selective HDAC inhibition with inhibitors suberoylanilide hydroxamic acid (SAHA), apicidin, MS-275, or MI-192 suppressed the expression of cytokines (IL-6 and TNF-α) in LPS-induced BV2 microglia (Durham et al., 2017). Unexpectedly, knockdown of HDAC1 resulted in an increase of HDAC2, while HDAC2 knockdown did not affect HDAC1 expression. And cells with HDAC1 silencing did not change IL-6 and TNF-α expression induced by LPS, while cells with HDAC2 downregulation notably decreased LPS-stimulated cytokines expression. This suggests that both HDACs redundantly regulates inflammatory response in microglia challenged by LPS (Durham et al., 2017). Although HDAC inhibitors SAHA and apicidin caused the upregulation of histone H4 acetylation in LPS-treated BV2 microglia, incubation of BV2 cells with cycloheximde to block new protein synthesis did not prevent either SAHA or apicidin inhibiting IL-6 expression. This indicates that the increase in protein expression due to enhanced histone H4 acetylation is not important for the reduction of microglial activation by HDAC inhibitors, and further investigations are needed (Durham et al., 2017). HDAC2 inhibitor CAY10683 could inhibit the expression levels of inflammatory cytokines TNF-α and IL-1β in LPS-activated BV2 microglial cells and mitigate LPS-induced neuroinflammation in mice (Jiao et al., 2018). LPS stimulation-induced the expression of TLR4, MyD88, phospho-NF-κB p65, and HDAC2 was decreased by CAY10683. Moreover, CAY10683 increased the acetylation of histone H3 in microglial cells (Jiao et al., 2018). These results indicate that CAY10683 suppresses TLR4/NF-κB signaling pathway in LPS-induced neuroinflammation mainly through the histones acetylation. The α7 nicotinic acetylcholine receptor (nAChR) plays a role in the neuroprotective function in microglia (Nakamura et al., 2020). LPS downregulates the expression of α7 nAChR mRNA through histone H3 deacetylation by HDAC2 and HDAC3 in BV2 cells (Nakamura et al., 2020). This suggests that histone H3 deacetylation at sites near the α7 nAChR promoter may be a mechanism through which microglial activation is induced. The inhibitory effect of LPS on α7 nAChR mRNA expression can be prevented by TLR4 blocker TAK-242, HDAC inhibitor trichostatin A, HDAC2/3 inhibitor MI-192, or selective HDAC3 inhibitor RGFP966 (Nakamura et al., 2020). These results suggest that LPS-TLR4 cascade activates HDAC kinases to suppress α7 nAChR mRNA expression in microglia (Figure 1).

#### 3.2.2 HDAC3

**FIGURE 2:**
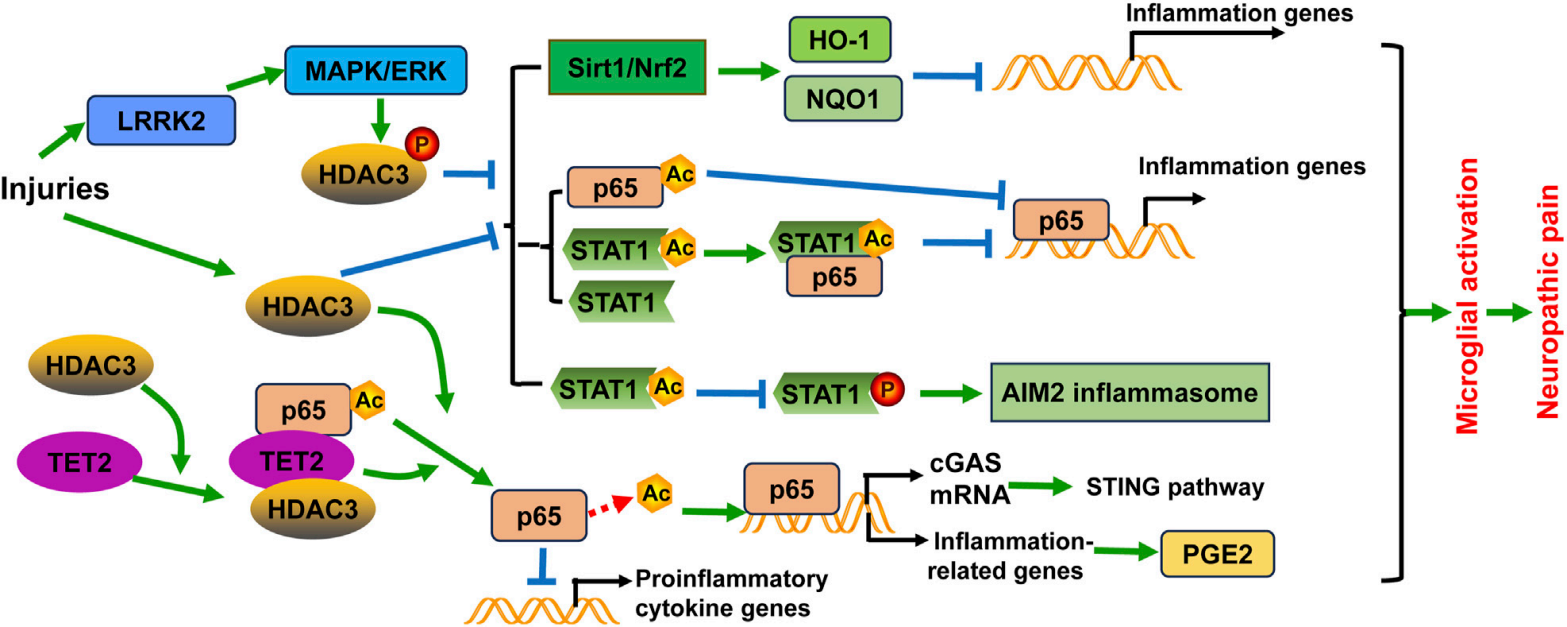
Schematic diagram of the potential mechanisms of HDAC3 in microglial activation and/or neuropathic pain. Injuries including spinal cord injury and ischemic stroke not only induce HDAC3 expression, but also promote HDAC3 activity through phosphorylation modification by the LRRK2/MAPK/ERK signaling pathway. HDAC3 expression and activity have several effects: (1) They inhibit the Sirt1/Nrf2 pathway to decrease the expression of heme oxygenase-1 (HO-1) and NAD(P)H quinone oxidoreductase 1 (NQO1), leading to the transcription of inflammation-related genes; (2) They decrease STAT1 expression and acetylation, as well as NF-κB p65 acetylation. Acetylated NF-κB p65 and the complex formed with acetylated STAT1 and NF-κB p65 both suppress the transcriptional activity of NF-κB p65, thereby reducing neuroinflammation; (3) They induce the formation of the AIM2 inflammasome through diminishing STAT1 acetylation and subsequently enhancing STAT1 tyrosine phosphorylation and activation in microglia. Furthermore, HDAC3 promotes NF-κB p65 deacetylation and nuclear localization, contributing to the activation of the cGAS-STING pathway and neuroinflammation. The deacetylation of NF-κB p65 also enhances the activation of downstream NF-κB target genes related to inflammation, such as Cox1 and Cox2, which are enzymes involved in PGE2 synthesis. Additionally, TET2 can recruit and interact with HDAC3 and acetylated NF-κB p65, leading to p65 deacetylation and consequently downregulating the transcription of proinflammatory cytokine genes in microglia.

The expression of HDAC3 is largely induced in microglia/macrophage following SCI and ischemic stroke (Kuboyama et al., 2017; Zhang et al., 2020). LRRK2 has been reported to regulate HDAC3 activity through phosphorylation modification via the MAPK/ERK signaling pathway, leading to alterations in inflammatory gene expression (Wei et al., 2022). Activation of overall reparative transcriptional profiles has been observed in injury-activated microglia and macrophages (IAM) upon SCI (Wahane et al., 2021). Importantly, most of the upstream transcriptional regulators of the IAM gene programs can be attributed to HDAC3 (Wahane et al., 2021). The distinct expression profiles of IAM have been revealed following SCI. Microglia express higher levels of genes associated with vascular endothelial growth factor (VEGF) signaling, cytokine response, IL-2 signaling, and TNF signaling, whereas macrophages exhibit higher levels of genes related to lysosome formation, IL-2 signaling and TGFβ regulation of ECM after SCI (Wahane et al., 2021). Similar to the roles of HDAC1 and HDAC2, selective blockade of HDAC3 by RGFP966 may reshape the phenotype of microglia/macrophage towards inflammatory suppression, thereby improving neurological functions, ameliorating Basso-Beattie-Bresnahan (BBB) permeability, and reducing brain edema following SCI (Kuboyama et al., 2017; Chen S. et al., 2023). As well, both RGFP966 and HDAC3 siRNA inhibits LPS-induced inflammatory activation of primary microglia (Kuboyama et al., 2017). RGFP966 also promotes Sirt1 expression and enhances Nrf2 nuclear accumulation and transcriptional activity, further activating the expression of activate heme oxygenase-1 (HO-1) and NAD(P)H quinone oxidoreductase 1 (NQO1) after SCI (Chen S. et al., 2023) (Figure 2). In another study, TBI upregulated HDAC1 and HDAC3 expression in the cortical tissues of model mice, with the activation of microglia and astrocytes. However, electroacupuncture (EA) could reverse HDAC1 and HDAC3 expression and alleviate function impairment in animal models (Hung et al., 2022). Microglia-specific HDAC3 knockout reduced proinflammatory microglia but augmented inflammation-resolving microglia after TBI. Moreover, HDAC3 knockout downregulated inflammatory cytokines in the brain while upregulated several anti-inflammatory cytokines. By remodeling microglial function, HDAC3 deficiency exerted long-term improvement of functional recovery after TBI (Zhao Y. et al., 2022). These results uncover that HDAC3 plays a critical part in the inflammatory response mediated by microglia after mechanical nerve injury.

Valproic acid (VPA) has long been used as an anticonvulsive drug, and recent research has unveiled its activity in inhibiting class I HDAC. VPA treatment has been shown to transform the M1 phenotype of microglia into the M2 phenotype and inhibit microglial activation, leading to the reduction of inflammatory response after SCI. In this process, the expression and activity of HDAC3 are decreased, while the expression and acetylation of STAT1, as well as NF-κB p65 acetylation are augmented by VPA (Chen S. et al., 2018). Notably, studies have found that acetylation of p65 at different sites can generate different effects, either activation or inactivation of p65 protein. Within the p65 protein, there are seven acetylated lysine residues, including K122, K123, K218, K221, K310, K314, and K315. Acetylation of K310 and K221 activates p65 and promotes NF-κB response, while acetylation of K122, K123, K218 impairs the NF-κB response. Acetylation of K314 and K315 in p65 differentially regulates the expression of specific sets of NF-κB target genes in response to TNF-α stimulation (Zhang et al., 2021). Acetylated NF-κB p65, along with the complex with NF-κB p65 and STAT1, suppresses the transcriptional activity of NF-κB p65, thereby reducing the inflammatory response following SCI (Chen S. et al., 2018). Furthermore, the regulation of NF-κB p65 acetylation and nuclear localization by HDAC3 contributes to the activation of the cGAS-STING pathway and neuroinflammation (Liao et al., 2020). TET2 is a member of the ten eleven translocation (TET) methylcytosine dioxygenase family. TET2 can recruit and interact with HDAC3 and acetylated NF-κB p65, leading to p65 deacetylation and thus downregulating the transcription of proinflammatory cytokine genes in microglia (Ma et al., 2021). Photothrombotic stroke and environmental stress lead to microglial overactivation in model mice, with an upregulation of HDAC3 expression in activated microglia. HDAC3 increases prostaglandin E2 (PGE2) production by activating the NF-κB signaling through p65 deacetylation (Zhu et al., 2022). These results strengthen the viewpoint that HDAC3 may act through p65 deacetylation to contribute to microglial activation. Likewise, VPA alleviated mechanical allodynia in rats induced by SNL. VPA suppresses neuroinflammation by reducing proinflammatory cytokines and increasing anti-inflammatory cytokines. It also inhibits spinal microgliosis and promotes the M2 phenotypic transition of microglia. These effects of VPA are dependent on the regulation of HDAC3 activity (Guo et al., 2021) (Figure 2). Notably, VPA and another HDAC inhibitor, sodium butyrate (NaBut), can promote the expression of prostaglandins (PGs) and the release of cytokines in microglia challenged by LPS *in vitro*, and induce histone acetylation at H3-K18 (Singh et al., 2014). However, VPA and NaBut did not affect the activation of p38, ERK1/2, and JNK/MAPKs signaling pathways after LPS treatment. This indicates that other factors may be involved in the participation of histone acetylation (Singh et al., 2014).

The study of a panel of novel HDAC3 inhibitors detailed the functions of HDAC3 in microglial activation. HDAC3-selective small-molecule inhibitor RGFP966 treatment protects against ischemic injury of mouse models by suppressing the formation of AIM2 inflammasome through enhancing STAT1 acetylation and subsequently diminishing STAT1 tyrosine phosphorylation and activation in microglia (Zhang et al., 2020) (Figure 2). BRD3308 (BRD) is an inhibitor of HDAC3 that negatively regulates inflammation-induced apoptosis. BRD treatment can alleviate neuronal function impairment, decrease neuronal loss, microglial activation, and pyroptosis in the hippocampus by activating the PPARγ/NLRP3/GSDMD signaling pathway in mice with intraventricular hemorrhage (Li Y. et al., 2023). This reveals the correlation between HDAC3 and pyroptosis in microglial activation and neuroinflammation (Li Y. et al., 2023). BG45 is a class Ⅰ HDAC inhibitor with selectivity of HDAC3. BG45 treatment decreased Aβ deposition, downregulated phosphorylation of tau protein, reduced IBA1-positive microglia and GFAP-positive astrocytes, suppressed the expression of inflammatory cytokines in the APP/PS1 mouse model of Alzheimer’s disease (Wang et al., 2023). These results suggest that different inhibitors may result in differential effects upon microglial activation.

#### 3.2.3 HDAC4

The expression of HDAC4 can be found in multiple tissues including brain, heart, colon, and so on (Wang et al., 1999). HDAC4 conditional knockout mice have been demonstrated to exhibit a reduction in thermal hypersensitivity in the complete Freund’s adjuvant model of inflammatory pain (Crow et al., 2015). HDAC4 expression could be induced by CCI in the dorsal root ganglion (DRG) of rats and involved in neuropathic pain (Wen et al., 2019). Targeting HDAC4 expression by miR-206-3p might alleviate neuropathic pain in animals (Wen et al., 2019). Moreover, in a neuropathic pain model established by SNL, HDAC4 phosphorylation was enhanced and cytoplasmic HDAC4 retention was promoted in the ipsilateral dorsal horn. This effect could be prevented by knockdown of spinal 14-3-3β expression, which was achieved by intrathecal injection of 14-3-3β siRNA into the dorsal subarachnoid space (L4 to L5) of rats. This indicated the modulation role of the 14-3-3β-HDAC4 axis in the development of neuropathic pain (Lin et al., 2015). IL-17A and activated microglia are critical factors inducing neurodevelopmental disorders in offspring. HDAC4 expression was found to be reduced in both IL-17A-treated primary microglia and in the fetal brains of LPS-induced maternal immune activation mouse model (Iitani et al., 2024). However, HDAC4 is required for efficient inflammatory cytokine production activated by LPS in BV2 microglia cells (Wang et al., 2014). Interestingly, prolonged LPS treatment activates GSK3β-iNOS-NO axis and caspase-3, which further cleaves HDAC4 protein and induces its degradation (Wang et al., 2014). This study implies a potential metabolism-based safeguard mechanism by which glycolysis not only drives the proinflammatory response, but also restricts the duration of inflammation by initiating the eventual degradation of HDAC4.

#### 3.2.4 HDAC6

CCI induced the enhancement of HDAC6 expression in the spinal cord, primarily expressed in neurons and microglia. The suppression of microglial activation via minocycline, a microglia inhibitor, suppressed HDAC6 expression after CCI (Sun et al., 2023). Furthermore, inhibition of HDAC6 by a specific inhibitor ACY-1215 alleviated mechanical allodynia, suppressed pyroptosis and neuroinflammation via activation of the NF-κB/NLRP3 pathway (Sun et al., 2023). However, high concentration of ACY-1215 also has activity at HDAC1, 2, and 3 (Santo et al., 2012). The specific inhibitor of HDAC6, PB131, not only suppressed the inflammatory response in LPS-treated BV2 microglia cells but also alleviated LPS-induced neuroinflammation in model mice (Liu et al., 2023). Sigma-1 receptor (Sig1R) is a pluripotent modulator protein involved in anti-inflammatory roles in microglia. LPS treatment could downregulate Sig1R expression and induce microglial activation via TLR4-TAK1-p38 MAPK pathway and the activation of HDAC6 in primary cultured microglia (Iwamoto et al., 2020). These studies suggest the promotive effect of HDAC6 in LPS-induced microglial activation.

#### 3.2.5 HDAC11

HDAC11 is abundantly expressed in the brain and plays a part in microglial activation. A HDAC11 inhibitor compound 5 has been developed to attenuate microglial activation by the initiation of autophagy and inhibition of nitric oxide production following LPS stimulation *in vitro* (Baek et al., 2023). Furthermore, HDAC11 inhibition could alleviate depression-like behavior in model mice (Baek et al., 2023). However, the effects of HDAC11 on neuropathic pain and related mechanisms need to be further clarified.

#### 3.2.6 Sirt1

Sirt1 is an essential member of the sirtuin family that exert deacetylase activity dependent on nicotinamide adenine dinucleotide (NAD^+^). Sirt1 plays important roles in neuropathic pain by regulating inflammatory response, oxidative stress, immune system, and epigenetic modification. The expression of Sirt1 within microglia cells is modulated by a variety of intracellular signaling pathways, including JNK (An et al., 2023), AMPK (Li et al., 2021), and PPARδ (Pan et al., 2024). In addition, Sirt1 expression may also be regulated by microRNAs, such as miR-210 and miR-155. The expression of these miRNAs may be enhanced in activated microglia following hypoxic-ischemic treatment (Li B. et al., 2020; Ke et al., 2023). Subsequently, the increased expression of miR-210 and miR-155 can target Sirt1 to regulate p65 deacetylation at lysine 310 and activation, which further contributes to microglial activation and neuroinflammation (Li B. et al., 2020; Ke et al., 2023). SRT2104 is an agonist of Sirt1 and can increase Sirt1 expression in somatic cells. Treatment of SRT2104 has been shown to alleviate oxygen glucose deprivation/reoxygenation (OGD/R)-induced neuronal and microglial death (Fu et al., 2021). Furthermore, SRT2104 can reverse OGD/R-induced microglial polarization by regulating the Sirt1/NF-κB signaling pathway (Fu et al., 2021). As well, Sirt1 can regulate the viability and apoptosis of microglia and suppress microglial activation by Sonic hedgehog (Shh)/GLI family zinc finger-1 (Gli-1) signaling following OGD/R injury *in vitro* (Liao et al., 2023). Inhibition of Sirt1 expression using its specific inhibitor EX527 resulted in microglial M1 polarization and activation of the NLRP3 inflammasome. Conversely, activation of Sirt1 with SRT1720 diminished oxidative stress and inflammatory response, and reshaped microglia polarization (Xia et al., 2021). SRT1720 also attenuated the detrimental effects of subarachnoid hemorrhage (SAH) on neuronal survival and function, which could be abated by EX527 (Xia et al., 2021). Collectively, these studies suggest that Sirt1 exerts a protective role in the alleviation of neuronal injury and neuroinflammation.

**FIGURE 3:**
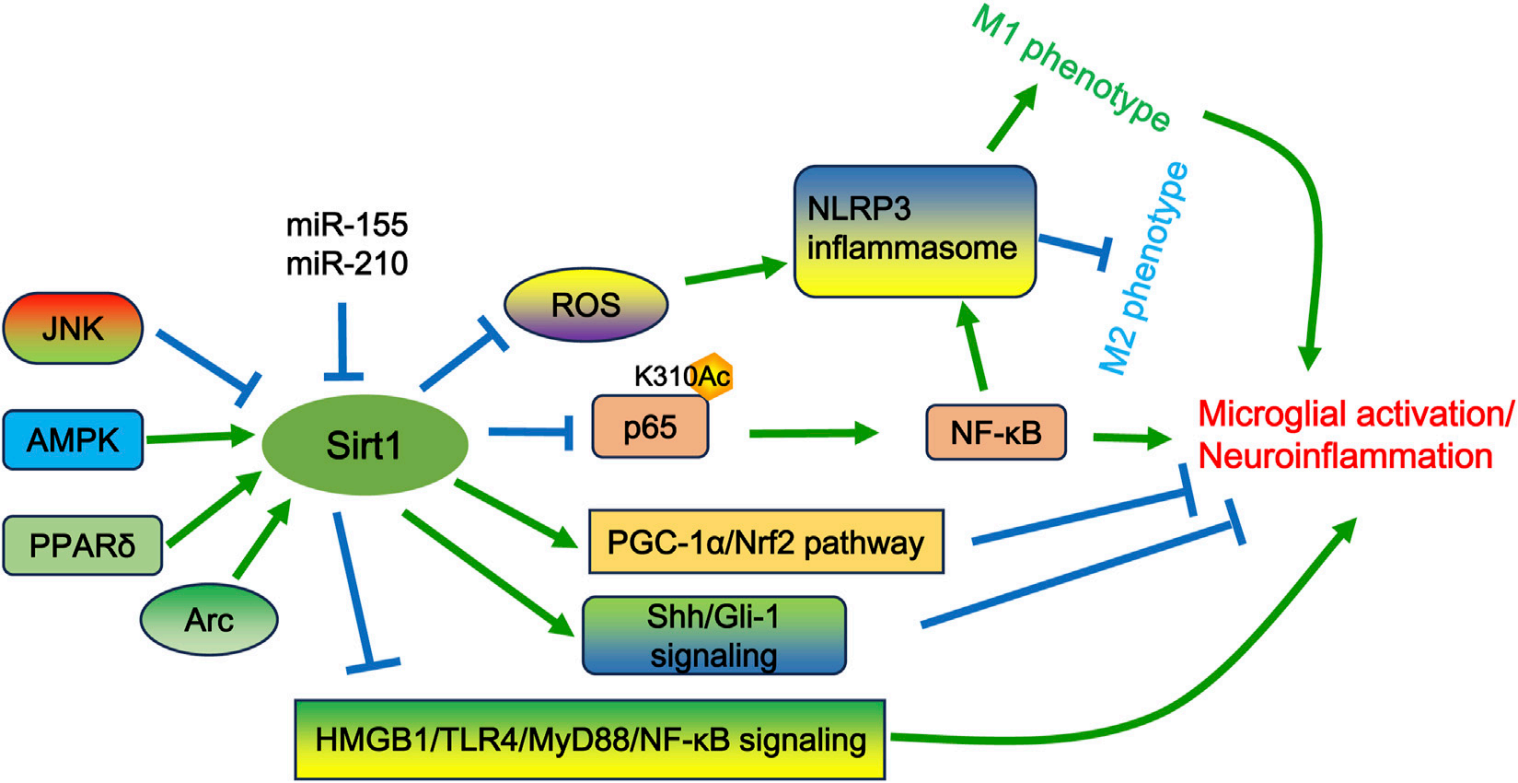
The potential mechanisms of Sirt1 in microglial activation and/or neuropathic pain. Sirt1 can be positively regulated by the AMPK and PPARδ pathways, as well as by Arc, but can be negatively regulated by JNK signaling and specific miRNAs (e.g., miR-210 and miR-155). Sirt1 governs excessive microglial activation by deacetylating p65 at lysine 310, thereby suppressing NF-κB activity. Additionally, Sirt1 can shift the microglial phenotype toward the M2 state by modulating ROS-mediated NLRP3 inflammasome signaling and the NF-κB/NLRP3 pathway. Furthermore, Sirt1 activates the PGC-1α/Nrf2 and Sonic hedgehog (Shh)/GLI family zinc finger-1 (Gli-1) signaling pathways to inhibit microglial activation. Additionally, Sirt1 can inhibit the release of HMGB1, which in turn suppresses the activation of the TLR4/MyD88/NF-κB signaling pathway.

At present, the development of many compounds that modulate Sirt1 activity is portraying the role of Sirt1 in microglial activation. 2,3,5,6-Tetramethylpyrazine (TMP), an active component of Ligusticum chuanxiong Hort, facilitates neurological recovery, attenuates the neuroinflammatory response, and prevents microglia M1 polarization following ischemia/reperfusion injury in LPS-induced neuroinflammation in mice through activating Sirt1. These effects can be reversed by EX527. TMP has also been shown to abate the neuroinflammatory response in LPS-treated BV2 cells by upregulating Sirt1 expression, which can be compromised by EX527 (Chen Y. et al., 2023). Omega-3 polyunsaturated fatty acids (ω-3 PUFA), which include eicosapentaenoic acid and docosahexaenoic acid, possess anti-oxidative and anti-inflammatory properties (Chen X. et al., 2018). ω-3 PUFA can elevate Sirt1 activity, thereby inhibiting HMGB1 acetylation in microglia. This action further promotes M2 microglial polarization and exerts neuroprotective effects after TBI (Chen X. et al., 2018). Inhibition of Sirt1 by sirtinol has been shown to promote microglial activation and increase the secretion of TNF-α, IL-6, and IL-1β following TBI (Chen et al., 2022). Sirt1 suppression also reduces the activation of the PGC-1α/Nrf2 pathway (Chen et al., 2022). In contrast, activation of Sirt1 by its activator A3 leads to the opposite effects (Chen et al., 2022). These results suggest that the PGC-1α/Nrf2 pathway is involved in the protective role of Sirt1 after TBI (Chen et al., 2022). Huangqi Guizhi Wuwu decoction (HGWD) exhibits neuroprotective effects in ischemic stroke, diabetic peripheral neuropathy, and oxaliplatin-induced peripheral neurotoxicity (Ou et al., 2023). The reduction of neurological deficits and neural loss by HGWD may be associated with its impact on microglial polarization through modification of the Sirt1/NF-κB/NLRP3 pathway (Ou et al., 2023). Nicotinamide n-oxide (NAMO), a product of the gut microbe *Lactobacillus*, has been implicated in the regulation of microglial activation (Li F. et al., 2022; Li F. et al., 2023). NAMO can alter the microglial phenotype from M1 to M2 and suppresses microglial inflammation following HSV-1 infection (Song et al., 2022). The effects of NAMO are correlated with its ability to activate Sirt1 and induce p65 deacetylation (Song et al., 2022). Melatonin can antagonize the effects of LPS on microglia and suppress the expression of proinflammatory cytokines (Chibaatar et al., 2021). The release of HMGB1, induced by LPS, results in neuroinflammation via the TLR4/MyD88/NF-κB signaling pathway. This neuroinflammatory response can be prevented by Sirt1 upregulation induced by melatonin pretreatment (Chibaatar et al., 2021). Pterostilbene (PTE), an analog of resveratrol, has been shown to protect the brain from injury caused by SAH and inhibit microglial activation via the Sirt1/Nrf2 signaling pathway (Zhang et al., 2022). Cycloastragenol (CAG) is an active component isolated from Astragalus Radix. CAG protects against ischemic brain injury by inducing Sirt1 expression and inhibiting the activation of microglia and astrocytes in the ischemic brain (Li M. et al., 2020). DJ-1 is a multifunctional protein involved in regulating oxidative stress, programmed cell death, and inflammation. Knockdown of DJ-1 shifts the phenotype of microglia from an anti-inflammatory phenotype to a proinflammatory state (Zhao N. et al., 2022). Importantly, inhibition of Sirt1 activity using EX-527 fortifies the effects of DJ-1 downregulation on microglial activation and constrains autophagy in the middle cerebral artery occlusion/reperfusion (MCAO/R) model (Zhao N. et al., 2022). Activity regulated cytoskeleton associated protein (Arc) is a postsynaptic protein implicated in synaptic plasticity. Knockdown of Arc exacerbates brain edema and oxidative stress, and augments the activation of microglia and astrocytes following SAH (Chen T. et al., 2023). However, activation of Sirt1 using the agonist SRT2104 markedly decreases brain damage and neuroinflammation induced by Arc knockdown (Chen T. et al., 2023). This indicates that Sirt1 can be positively regulated by Arc (Figure 3).

#### 3.2.7 Sirt2

The absence of Sirt2 leads to morphological changes in microglia and an increase in the expression of proinflammatory cytokines upon LPS treatment (Pais et al., 2013). Sirt2 overexpression inhibits microglial responses, whereas Sirt2 knockdown promotes them (Pais et al., 2013). Importantly, its deacetylase activity can be diminished by phosphorylation at S331 (Pais et al., 2013). Sirt2 deletion also leads to NF-κB hyperacetylation, which contributes to excessive microglial activation (Pais et al., 2013). Remifentanil (Remi) is an ultra-fast-acting opioid drug frequently used in clinical anesthesia. However, Remil-induced hyperalgesia also brings a burden on patients with unclear mechanisms. Remi treatment activates microglia and downregulates Sirt2 expression in the spinal cord (Zhu and Ma, 2023). Sirt2 overexpression relieves Remi-induced hyperalgesia and mechanical allodynia by inhibiting microglial activation (Zhu and Ma, 2023).

#### 3.2.8 Sirt3

Sirt3 expression is significantly correlated with microglial activation and is critical for the protection of neuronal function after SAH (Liu et al., 2018; Guo et al., 2023). P7C3-A20, an aminopropyl carbazole compound, can promote the NAD^+^ salvage pathway by interacting with nicotinamide phosphoribosyl transferase (NAMPT). P7C3-A20 reduces brain lesion, promotes neuronal function recovery, ameliorates BBB impairment and edema, and suppresses microglial activation and neuroinflammation in a Sirt3-dependent manner (Wu et al., 2023). Sirt3 expression is reduced during the activation of microglia by LPS, Sirt3 overexpression alleviates LPS-induced BV2 cell death (Zhou and Jiang, 2019). Sirt3 functions by maintaining normal mitochondrial function and decreasing mitochondria dependent apoptosis by the Mst1-JNK pathway (Zhou and Jiang, 2019). Triggering receptor expressed on myeloid cells 2 (TREM2) is involved in the *de novo* synthesis pathway of NAD^+^, which is important for the activity of Sirt3 and other Class III HDACs (Li H. et al., 2022). TREM2 overexpression attenuates oxidative stress and neuroinflammation in BV2 cells by enhancing Sirt3 function (Li H. et al., 2022).

## 4 Conclusion and future perspectives

As the resident macrophages of the CNS, microglia play a pivotal role in pain modulation. Their activation not only sensitizes nociceptors and enhances their responsiveness to noxious stimuli through the release of proinflammatory cytokines and chemokines, but also influences pain perception and modulation through interaction with the immune system. Recent studies have demonstrated that the expression levels and/or activities of HDAC1, HDAC2, HDAC3, and HDAC6 are upregulated during injury. This upregulation can promote microglial activation and neuropathic pain, with HDAC2 exhibiting a distinct role in microglial activation across different neuropathic pain animal models. Sirt1, Sirt2, and Sirt3 may exhibit protective effects against neuropathic pain because they can inhibit microglial activation. However, the role of HDAC4 in neuropathic pain models appears to be contradictory. HDAC4 not only acts as a promoter of proinflammatory cytokines but also serves as a regulator of the immunometabolic program in microglia (Crow et al., 2015; Wen et al., 2019; Iitani et al., 2024; Wang et al., 2014). Although HDAC11 inhibitor has been found to attenuate microglial activation and alleviate depression-like behavior, the specific roles and mechanisms of HDAC11 in microglial activation and neuropathic pain remain unclear. Notably, there are fewer reports on the effects of other HDACs, such as HDAC5, HDAC7, HDAC8, HDAC9, HDAC10, Sirt4, Sirt5, Sirt6, and Sirt7, in microglial activation and neuropathic pain. These areas require further study in the future.

A growing body of evidence indicates that HDAC inhibitors or activators have potential therapeutic effects on multiple diseases, including neurological diseases (Zhang L. Y. et al., 2024; Razick et al., 2023). Similarly, current studies have found that the application of HDAC inhibitors or activators can also suppress microglial activation and alleviate neuropathic pain in preclinical models. However, given the numerous subtypes of HDACs and the significant similarities in their active domains and catalytic sites, achieving the development of HDAC inhibitors with high subtype selectivity represents a future breakthrough point that is likely to encounter substantial challenges in practical research (Zhang L. Y. et al., 2024). It is necessary to test their efficacy in clinical practice. In the future, as more functions of HDACs in microglial activation are clarified, and more efficient and accurate HDAC inhibitors or activators are developed, the treatment of neuropathic pain will become a practical possibility.

## References

[B1] An M. Qiu Y. Wang C. Ma P. Ding Y. (2023). Rac2 enhances activation of microglia and astrocytes, inflammatory response, and apoptosis via activating JNK signaling pathway and suppressing SIRT1 expression in chronic constriction injury-induced neuropathic pain. J. Neuropathol. Exp. Neurol. 82 (5), 419–426. 10.1093/jnen/nlad006 36779914

[B2] Atta A. A. Ibrahim W. W. Mohamed A. F. Abdelkader N. F. (2023). Microglia polarization in nociplastic pain: mechanisms and perspectives. Inflammopharmacology 31 (3), 1053–1067. 10.1007/s10787-023-01216-x 37069462 PMC10229465

[B3] Auzmendi-Iriarte J. Moreno-Cugnon L. Saenz-Antoñanzas A. Grassi D. de Pancorbo M. M. Arevalo M. A. (2022). High levels of HDAC expression correlate with microglial aging. Expert Opin. Ther. Targets 26 (10), 911–922. 10.1080/14728222.2022.2158081 36503367

[B4] Baek S. Y. Lee J. Kim T. Lee H. Choi H. S. Park H. (2023). Development of a novel histone deacetylase inhibitor unveils the role of HDAC11 in alleviating depression by inhibition of microglial activation. Biomed. Pharmacother. 166, 115312. 10.1016/j.biopha.2023.115312 37567072

[B5] Basic-Kes V. Zavoreo I. Bosnar-Puretić M. Ivanković M. Bitunjac M. Govori V. (2009). Neuropathic pain. Acta Clin. Croat. 48 (3), 359–365. 20055264

[B6] Bellver-Landete V. Bretheau F. Mailhot B. Vallières N. Lessard M. Janelle M. E. (2019). Microglia are an essential component of the neuroprotective scar that forms after spinal cord injury. Nat. Commun. 10 (1), 518. 10.1038/s41467-019-08446-0 30705270 PMC6355913

[B7] Bennett G. J. (2012). What is spontaneous pain and who has it? J. Pain 13 (10), 921–929. 10.1016/j.jpain.2012.05.008 22824584

[B8] Borgonetti V. Governa P. Manetti F. Galeotti N. (2023). Zingiberene, a non-zinc-binding class I HDAC inhibitor: A novel strategy for the management of neuropathic pain. Phytomedicine 111, 154670. 10.1016/j.phymed.2023.154670 36681053

[B9] Carroll J. A. Chesebro B. (2019). Neuroinflammation, microglia, and cell-Association during prion disease. Viruses 11 (1), 65. 10.3390/v11010065 30650564 PMC6356204

[B10] Casaril A. M. Gaffney C. M. Shepherd A. J. (2024). Animal models of neuropathic pain. Int. Rev. Neurobiol. 179, 339–401. 10.1016/bs.irn.2024.10.004 39580217

[B11] Cavalli E. Mammana S. Nicoletti F. Bramanti P. Mazzon E. (2019). The neuropathic pain: An overview of the current treatment and future therapeutic approaches. Int. J. Immunopathol. Pharmacol. 33, 2058738419838383. 10.1177/2058738419838383 30900486 PMC6431761

[B12] Celik M. O. Labuz D. Keye J. Glauben R. Machelska H. (2020). IL-4 induces M2 macrophages to produce sustained analgesia via opioids. JCI Insight 5 (4), e133093. 10.1172/jci.insight.133093 32102987 PMC7101153

[B13] Challa S. R. (2015). Surgical animal models of neuropathic pain: Pros and Cons. Int. J. Neurosci. 125 (3), 170–174. 10.3109/00207454.2014.922559 24831263

[B14] Chen S. Ye J. Chen X. Shi J. Wu W. Lin W. (2018a). Valproic acid attenuates traumatic spinal cord injury-induced inflammation via STAT1 and NF-κB pathway dependent of HDAC3. J. Neuroinflammation 15 (1), 150. 10.1186/s12974-018-1193-6 29776446 PMC5960086

[B15] Chen S. Ye J. Wu G. Shi J. Li X. Chen X. (2023a). Histone deacetylase 3 inhibition ameliorates microglia-mediated neuro-inflammation via the SIRT1/Nrf2 pathway after traumatic spinal cord injury. Neurorehabil Neural Repair 37 (8), 503–518. 10.1177/15459683231183716 37503724

[B16] Chen T. Xu Y. P. Chen Y. Sun S. Yan Z. Z. Wang Y. H. (2023c). Arc regulates brain damage and neuroinflammation via Sirt1 signaling following subarachnoid hemorrhage. Brain Res. Bull. 203, 110780. 10.1016/j.brainresbull.2023.110780 37820952

[B17] Chen X. Chen C. Fan S. Wu S. Yang F. Fang Z. (2018b). Omega-3 polyunsaturated fatty acid attenuates the inflammatory response by modulating microglia polarization through SIRT1-mediated deacetylation of the HMGB1/NF-κB pathway following experimental traumatic brain injury. J. Neuroinflammation 15 (1), 116. 10.1186/s12974-018-1151-3 29678169 PMC5909267

[B18] Chen X. Wei G. Li D. Fan Y. Zeng Y. Qian Z. (2022). Sirtuin 1 alleviates microglia-induced inflammation by modulating the PGC-1α/Nrf2 pathway after traumatic brain injury in male rats. Brain Res. Bull. 185, 28–38. 10.1016/j.brainresbull.2022.04.012 35487384

[B19] Chen Y. Peng F. Yang C. Hou H. Xing Z. Chen J. (2023b). SIRT1 activation by 2,3,5,6-tetramethylpyrazine alleviates neuroinflammation via inhibiting M1 microglia polarization. Front. Immunol. 14, 1206513. 10.3389/fimmu.2023.1206513 37600790 PMC10436537

[B20] Chibaatar E. Le K. Abdoulaye I. A. Wu S. Guo Y. (2021). Melatonin ameliorates lipopolysaccharide-induced microglial inflammation via triggering SIRT1/HMGB1 signaling Axis. J. Mol. Neurosci. 71 (4), 691–701. 10.1007/s12031-020-01699-1 32910356

[B21] Cornell J. Salinas S. Huang H. Y. Zhou M. (2022). Microglia regulation of synaptic plasticity and learning and memory. Neural Regen. Res. 17 (4), 705–716. 10.4103/1673-5374.322423 34472455 PMC8530121

[B22] Crow M. Khovanov N. Kelleher J. H. Sharma S. Grant A. D. Bogdanov Y. (2015). HDAC4 is required for inflammation-associated thermal hypersensitivity. FASEB J. 29 (8), 3370–3378. 10.1096/fj.14-264440 25903105 PMC4511203

[B23] Datta M. Staszewski O. Raschi E. Frosch M. Hagemeyer N. Tay T. L. (2018). Histone deacetylases 1 and 2 regulate microglia function during development, homeostasis, and neurodegeneration in a context-dependent manner. Immunity 48 (3), 514–529 e6. 10.1016/j.immuni.2018.02.016 29548672

[B24] Durham B. S. Grigg R. Wood I. C. (2017). Inhibition of histone deacetylase 1 or 2 reduces induced cytokine expression in microglia through a protein synthesis independent mechanism. J. Neurochem. 143 (2), 214–224. 10.1111/jnc.14144 28796285

[B25] Fornasari D. (2012). Pain mechanisms in patients with chronic pain. Clin. Drug Investig. 32 (Suppl. 1), 45–52. 10.2165/11630070-000000000-00000 22356223

[B26] Fu C. Y. Zhong C. R. Yang Y. T. Zhang M. Li W. A. Zhou Q. (2021). Sirt1 activator SRT2104 protects against oxygen-glucose deprivation/reoxygenation-induced injury via regulating microglia polarization by modulating Sirt1/NF-κB pathway. Brain Res. 1753, 147236. 10.1016/j.brainres.2020.147236 33412146

[B27] Giblin S. P. Midwood K. S. (2015). Tenascin-C: Form versus function. Cell Adh Migr. 9 (1-2), 48–82. 10.4161/19336918.2014.987587 25482829 PMC4422809

[B28] Guo A. Li J. Luo L. Chen C. Lu Q. Ke J. (2021). Valproic acid mitigates spinal nerve ligation-induced neuropathic pain in rats by modulating microglial function and inhibiting neuroinflammatory response. Int. Immunopharmacol. 92, 107332. 10.1016/j.intimp.2020.107332 33421931

[B29] Guo X. Chen D. An S. Wang Z. (2020). ChIP-seq profiling identifies histone deacetylase 2 targeting genes involved in immune and inflammatory regulation induced by calcitonin gene-related peptide in microglial cells. J. Immunol. Res. 2020, 4384696. 10.1155/2020/4384696 32832570 PMC7424498

[B30] Guo Y. Hu Y. Huang Y. Huang L. Kanamaru H. Takemoto Y. (2023). Role of estrogen-related receptor γ and PGC-1α/SIRT3 pathway in early brain injury after subarachnoid hemorrhage. Neurotherapeutics 20 (3), 822–837. 10.1007/s13311-022-01330-8 36481985 PMC10275823

[B31] Haage V. Elmadany N. Roll L. Faissner A. Gutmann D. H. Semtner M. (2019). Tenascin C regulates multiple microglial functions involving TLR4 signaling and HDAC1. Brain Behav. Immun. 81, 470–483. 10.1016/j.bbi.2019.06.047 31271872

[B32] Han Z. Zhao H. Tao Z. Wang R. Fan Z. Luo Y. (2018). TOPK promotes microglia/macrophage polarization towards M2 phenotype via inhibition of HDAC1 and HDAC2 activity after transient cerebral ischemia. Aging Dis. 9 (2), 235–248. 10.14336/AD.2017.0328 29896413 PMC5963345

[B33] Hua T. Kong E. Zhang H. Lu J. Huang K. Ding R. (2024). PRMT6 deficiency or inhibition alleviates neuropathic pain by decreasing glycolysis and inflammation in microglia. Brain Behav. Immun. 118, 101–114. 10.1016/j.bbi.2024.02.027 38402915

[B34] Hung S. Y. Chung H. Y. Luo S. T. Chu Y. T. Chen Y. H. MacDonald I. J. (2022). Electroacupuncture improves TBI dysfunction by targeting HDAC overexpression and BDNF-associated Akt/GSK-3β signaling. Front. Cell Neurosci. 16, 880267. 10.3389/fncel.2022.880267 36016833 PMC9396337

[B35] Iitani Y. Miki R. Imai K. Fuma K. Ushida T. Tano S. (2024). Interleukin-17A stimulation induces alterations in Microglial microRNA expression profiles. Pediatr. Res. 95 (1), 167–173. 10.1038/s41390-023-02825-6 37758861

[B36] Inoue K. Tsuda M. (2018). Microglia in neuropathic pain: cellular and molecular mechanisms and therapeutic potential. Nat. Rev. Neurosci. 19 (3), 138–152. 10.1038/nrn.2018.2 29416128

[B37] Iwamoto M. Nakamura Y. Takemura M. Hisaoka-Nakashima K. Morioka N. (2020). TLR4-TAK1-p38 MAPK pathway and HDAC6 regulate the expression of sigma-1 receptors in rat primary cultured microglia. J. Pharmacol. Sci. 144 (1), 23–29. 10.1016/j.jphs.2020.06.007 32653342

[B38] Jaggi A. S. Jain V. Singh N. (2011). Animal models of neuropathic pain. Fundam. Clin. Pharmacol. 25 (1), 1–28. 10.1111/j.1472-8206.2009.00801.x 20030738

[B39] Jensen T. S. Finnerup N. B. (2014). Allodynia and hyperalgesia in neuropathic pain: clinical manifestations and mechanisms. Lancet Neurol. 13 (9), 924–935. 10.1016/S1474-4422(14)70102-4 25142459

[B40] Jiao F. Z. Wang Y. Zhang H. Y. Zhang W. B. Wang L. W. Gong Z. J. (2018). Histone deacetylase 2 inhibitor CAY10683 alleviates lipopolysaccharide induced neuroinflammation through attenuating TLR4/NF-κB signaling pathway. Neurochem. Res. 43 (6), 1161–1170. 10.1007/s11064-018-2532-9 29675728

[B41] Jo K. W. Lee D. Cha D. G. Oh E. Choi Y. H. Kim S. (2022). Gossypetin ameliorates 5xFAD spatial learning and memory through enhanced phagocytosis against Aβ. Alzheimers Res. Ther. 14 (1), 158. 10.1186/s13195-022-01096-3 36271414 PMC9585741

[B42] Kami K. Taguchi S. Tajima F. Senba E. (2016). Histone acetylation in microglia contributes to exercise-induced hypoalgesia in neuropathic pain model mice. J. Pain 17 (5), 588–599. 10.1016/j.jpain.2016.01.471 26844418

[B43] Karavis M. Y. Siafaka I. Vadalouca A. Georgoudis G. (2023). Role of microglia in neuropathic pain. Cureus 15 (8), e43555. 10.7759/cureus.43555 37719474 PMC10503876

[B44] Ke F. Wang H. Geng J. Jing X. Fang F. Fang C. (2023). MiR-155 promotes inflammation and apoptosis via targeting SIRT1 in hypoxic-ischemic brain damage. Exp. Neurol. 362, 114317. 10.1016/j.expneurol.2023.114317 36608839

[B45] Kerstman E. Ahn S. Battu S. Tariq S. Grabois M. (2013). Neuropathic pain. Handb. Clin. Neurol. 110, 175–187. 10.1016/B978-0-444-52901-5.00015-0 23312640

[B46] Kettenmann H. Kirchhoff F. Verkhratsky A. (2013). Microglia: new roles for the synaptic stripper. Neuron 77 (1), 10–18. 10.1016/j.neuron.2012.12.023 23312512

[B47] Kohno K. Shirasaka R. Yoshihara K. Mikuriya S. Tanaka K. Takanami K. (2022). A spinal microglia population involved in remitting and relapsing neuropathic pain. Science 376 (6588), 86–90. 10.1126/science.abf6805 35357926

[B48] Kuboyama T. Wahane S. Huang Y. Zhou X. Wong J. K. Koemeter-Cox A. (2017). HDAC3 inhibition ameliorates spinal cord injury by immunomodulation. Sci. Rep. 7 (1), 8641. 10.1038/s41598-017-08535-4 28819194 PMC5561061

[B49] Kulthinee S. Yano N. Zhuang S. Wang L. Zhao T. C. (2022). Critical functions of histone deacetylases (HDACs) in modulating inflammation associated with cardiovascular diseases. Pathophysiology 29 (3), 471–485. 10.3390/pathophysiology29030038 35997393 PMC9397025

[B50] Li B. Dasgupta C. Huang L. Meng X. Zhang L. (2020a). MiRNA-210 induces microglial activation and regulates microglia-mediated neuroinflammation in neonatal hypoxic-ischemic encephalopathy. Cell Mol. Immunol. 17 (9), 976–991. 10.1038/s41423-019-0257-6 31300734 PMC7608107

[B51] Li D. Xu J. Qin Y. Cai N. Cheng Y. Wang H. (2021). Roflupram, a novel phosphodiesterase 4 inhibitor, inhibits lipopolysaccharide-induced neuroinflammatory responses through activation of the AMPK/Sirt1 pathway. Int. Immunopharmacol. 90, 107176. 10.1016/j.intimp.2020.107176 33243606

[B52] Li F. Wang Y. Song X. Wang Z. Jia J. Qing S. (2022a). The intestinal microbial metabolite nicotinamide n-oxide prevents herpes simplex encephalitis via activating mitophagy in microglia. Gut Microbes 14 (1), 2096989. 10.1080/19490976.2022.2096989 35793266 PMC9262364

[B53] Li F. Wang Y. Zheng K. (2023c). Microglial mitophagy integrates the microbiota-gut-brain axis to restrain neuroinflammation during neurotropic herpesvirus infection. Autophagy 19 (2), 734–736. 10.1080/15548627.2022.2102309 35849507 PMC9851194

[B54] Li H. Liu F. Jiang W. Wang K. Cao X. Zou J. (2022b). TREM2 ameliorates lipopolysaccharide-induced oxidative stress response and neuroinflammation by promoting Sirtuin3 in BV2 cells. Neurotox. Res. 40 (1), 56–65. 10.1007/s12640-021-00459-2 35013907

[B55] Li M. Li S. C. Dou B. K. Zou Y. X. Han H. Z. Liu D. X. (2020b). Cycloastragenol upregulates SIRT1 expression, attenuates apoptosis and suppresses neuroinflammation after brain ischemia. Acta Pharmacol. Sin. 41 (8), 1025–1032. 10.1038/s41401-020-0386-6 32203080 PMC7471431

[B56] Li X. Shi H. Zhang D. Jing B. Chen Z. Zheng Y. (2023a). Paeonol alleviates neuropathic pain by modulating microglial M1 and M2 polarization via the RhoA/p38MAPK signaling pathway. CNS Neurosci. Ther. 29 (9), 2666–2679. 10.1111/cns.14211 37032648 PMC10401133

[B57] Li Y. Liu C. Wang G. Wang H. Liu X. Huang C. (2023b). HDAC3 inhibitor (BRD3308) modulates microglial pyroptosis and neuroinflammation through PPARγ/NLRP3/GSDMD to improve neurological function after intraventricular hemorrhage in mice. Neuropharmacology 237, 109633. 10.1016/j.neuropharm.2023.109633 37327970

[B58] Liao H. Huang J. Liu J. Zhu H. Chen Y. Li X. (2023). Sirt1 regulates microglial activation and inflammation following oxygen-glucose deprivation/reoxygenation injury by targeting the Shh/Gli-1 signaling pathway. Mol. Biol. Rep. 50 (4), 3317–3327. 10.1007/s11033-022-08167-6 36725745 PMC10042964

[B59] Liao Y. Cheng J. Kong X. Li S. Li X. Zhang M. (2020). HDAC3 inhibition ameliorates ischemia/reperfusion-induced brain injury by regulating the microglial cGAS-STING pathway. Theranostics 10 (21), 9644–9662. 10.7150/thno.47651 32863951 PMC7449914

[B60] Lin T. B. Hsieh M. C. Lai C. Y. Cheng J. K. Chau Y. P. Ruan T. (2015). Modulation of nerve injury-induced HDAC4 cytoplasmic retention contributes to neuropathic pain in rats. Anesthesiology 123 (1), 199–212. 10.1097/ALN.0000000000000663 25871743

[B61] Liu G. Mondal P. Sang N. Li Z. Ding W. Yang L. (2023). Design, synthesis, and anti-inflammatory activity characterization of novel brain-permeable HDAC6 inhibitors. Eur. J. Med. Chem. 254, 115327. 10.1016/j.ejmech.2023.115327 37098307

[B62] Liu S. J. Liu X. Y. Li J. H. Guo J. Li F. Gui Y. (2018). Gastrodin attenuates microglia activation through renin-angiotensin system and Sirtuin3 pathway. Neurochem. Int. 120, 49–63. 10.1016/j.neuint.2018.07.012 30075231

[B63] Luan Y. Liu H. Yang Y. Yang J. Ren K. D. (2022). New insight in HDACs: potential therapeutic targets for the treatment of atherosclerosis. Front. Pharmacol. 13, 863677. 10.3389/fphar.2022.863677 35529430 PMC9068932

[B64] Luo L. Martin S. C. Parkington J. Cadena S. M. Zhu J. Ibebunjo C. (2019). HDAC4 controls muscle homeostasis through deacetylation of myosin heavy chain, PGC-1α, and Hsc70. Cell Rep. 29 (3), 749–763 e12. 10.1016/j.celrep.2019.09.023 31618641

[B65] Ma Q. Dasgupta C. Shen G. Li Y. Zhang L. (2021). MicroRNA-210 downregulates TET2 and contributes to inflammatory response in neonatal hypoxic-ischemic brain injury. J. Neuroinflammation 18 (1), 6. 10.1186/s12974-020-02068-w 33402183 PMC7786974

[B66] Miao J. Chen Z. Wu Y. Hu Q. Ji T. (2022). Sp1 inhibits PGC-1α via HDAC2-catalyzed histone deacetylation in chronic constriction injury-induced neuropathic pain. ACS Chem. Neurosci. 13 (23), 3438–3452. 10.1021/acschemneuro.2c00440 36401579

[B67] Nakamura Y. Kimura S. Takada N. Takemura M. Iwamoto M. Hisaoka-Nakashima K. (2020). Stimulation of toll-like receptor 4 downregulates the expression of α7 nicotinic acetylcholine receptors via histone deacetylase in rodent microglia. Neurochem. Int. 138, 104751. 10.1016/j.neuint.2020.104751 32413437

[B68] Ou Z. Zhao M. Xu Y. Wu Y. Qin L. Fang L. (2023). Huangqi Guizhi Wuwu decoction promotes M2 microglia polarization and synaptic plasticity via Sirt1/NF-κB/NLRP3 pathway in MCAO rats. Aging (Albany NY) 15 (19), 10031–10056. 10.18632/aging.204989 37650573 PMC10599726

[B69] Pais T. F. Szegő É. M. Marques O. Miller-Fleming L. Antas P. Guerreiro P. (2013). The NAD-dependent deacetylase sirtuin 2 is a suppressor of microglial activation and brain inflammation. EMBO J. 32 (19), 2603–2616. 10.1038/emboj.2013.200 24013120 PMC3791374

[B70] Pan H. C. Yang C. N. Lee W. J. Sheehan J. Wu S. M. Chen H. S. (2024). Melatonin enhanced microglia M2 polarization in rat model of neuro-inflammation via regulating ER stress/PPARδ/SIRT1 signaling Axis. J. Neuroimmune Pharmacol. 19 (1), 11. 10.1007/s11481-024-10108-y 38530514

[B71] Ponomarev E. D. Veremeyko T. Weiner H. L. (2013). MicroRNAs are universal regulators of differentiation, activation, and polarization of microglia and macrophages in normal and diseased CNS. Glia 61 (1), 91–103. 10.1002/glia.22363 22653784 PMC3434289

[B72] Razick D. I. Akhtar M. Wen J. Alam M. Dean N. Karabala M. (2023). The role of sirtuin 1 (SIRT1) in neurodegeneration. Cureus 15 (6), e40463. 10.7759/cureus.40463 37456463 PMC10349546

[B73] Santo L. Hideshima T. Kung A. L. Tseng J. C. Tamang D. Yang M. (2012). Preclinical activity, pharmacodynamic, and pharmacokinetic properties of a selective HDAC6 inhibitor, ACY-1215, in combination with bortezomib in multiple myeloma. Blood 119 (11), 2579–2589. 10.1182/blood-2011-10-387365 22262760 PMC3337713

[B74] Scholz J. Finnerup N. B. Attal N. Aziz Q. Baron R. Bennett M. I. (2019). The IASP classification of chronic pain for ICD-11: chronic neuropathic pain. Pain 160 (1), 53–59. 10.1097/j.pain.0000000000001365 30586071 PMC6310153

[B75] Singh V. Bhatia H. S. Kumar A. de Oliveira A. C. P. Fiebich B. L. (2014). Histone deacetylase inhibitors valproic acid and sodium butyrate enhance prostaglandins release in lipopolysaccharide-activated primary microglia. Neuroscience 265, 147–157. 10.1016/j.neuroscience.2014.01.037 24480366

[B76] Song X. Cao W. Wang Z. Li F. Xiao J. Zeng Q. (2022). Nicotinamide n-oxide attenuates HSV-1-Induced microglial inflammation through sirtuin-1/NF-κB signaling. Int. J. Mol. Sci. 23 (24), 16085. 10.3390/ijms232416085 36555725 PMC9784159

[B77] Stonedahl S. Clarke P. Tyler K. L. (2020). The role of microglia during West Nile virus infection of the central nervous system. Vaccines (Basel) 8 (3), 485. 10.3390/vaccines8030485 32872152 PMC7563127

[B78] Sun K. Zhang H. Zhang T. Sun N. Hao J. Wang Z. (2023). Spinal HDAC6 mediates nociceptive behaviors induced by chronic constriction injury via neuronal activation and neuroinflammation. Mol. Pain 19, 17448069231218352. 10.1177/17448069231218352 37982151 PMC10734332

[B79] Szczudlik A. Dobrogowski J. Wordliczek J. Stępień A. Krajnik M. Leppert W. (2014). Diagnosis and management of neuropathic pain: review of literature and recommendations of the Polish Association for the Study of Pain and the Polish Neurological Society - Part Two. Neurol. Neurochir. Pol. 48 (6), 423–435. 10.1016/j.pjnns.2014.11.002 25482254

[B80] Tozaki-Saitoh H. Tsuda M. (2019). Microglia-neuron interactions in the models of neuropathic pain. Biochem. Pharmacol. 169, 113614. 10.1016/j.bcp.2019.08.016 31445020

[B81] Tsuda M. (2019). Microglia-mediated regulation of neuropathic pain: Molecular and cellular mechanisms. Biol. Pharm. Bull. 42 (12), 1959–1968. 10.1248/bpb.b19-00715 31787711

[B82] Tu H. Chu H. Guan S. Hao F. Xu N. Zhao Z. (2021). The role of the M1/M2 microglia in the process from cancer pain to morphine tolerance. Tissue Cell 68, 101438. 10.1016/j.tice.2020.101438 33220596

[B83] Tu Y. Muley M. M. Beggs S. Salter M. W. (2022). Microglia-independent peripheral neuropathic pain in male and female mice. Pain 163 (11), e1129–e1144. 10.1097/j.pain.0000000000002643 35384869 PMC9578531

[B84] Wahane S. Zhou X. Zhou X. Guo L. Friedl M. S. Kluge M. (2021). Diversified transcriptional responses of myeloid and glial cells in spinal cord injury shaped by HDAC3 activity. Sci. Adv. 7 (9), eabd8811. 10.1126/sciadv.abd8811 33637528 PMC7909890

[B85] Wang A. H. Bertos N. R. Vezmar M. Pelletier N. Crosato M. Heng H. H. (1999). HDAC4, a human histone deacetylase related to yeast HDA1, is a transcriptional corepressor. Mol. Cell Biol. 19 (11), 7816–7827. 10.1128/MCB.19.11.7816 10523670 PMC84849

[B86] Wang B. Liu T. Y. Lai C. H. Rao Y. h. Choi M. C. Chi J. T. (2014). Glycolysis-dependent histone deacetylase 4 degradation regulates inflammatory cytokine production. Mol. Biol. Cell 25 (21), 3300–3307. 10.1091/mbc.E13-12-0757 25187650 PMC4214777

[B87] Wang C. Shen D. Hu Y. Chen J. Liu J. Huang Y. (2023). Selective targeting of class I HDAC reduces microglial inflammation in the entorhinal cortex of young APP/PS1 mice. Int. J. Mol. Sci. 24 (5), 4805. 10.3390/ijms24054805 36902234 PMC10003411

[B88] Wang J. Zhao H. Fan Z. Li G. Ma Q. Tao Z. (2017). Long noncoding RNA H19 promotes neuroinflammation in ischemic stroke by driving histone deacetylase 1-dependent M1 microglial polarization. Stroke 48 (8), 2211–2221. 10.1161/STROKEAHA.117.017387 28630232

[B89] Wei W. Liu Y. Qiu Y. Chen M. Wang Y. Han Z. (2022). Characterization of acetylation of histone H3 at lysine 9 in the trigeminal ganglion of a rat trigeminal neuralgia model. Oxid. Med. Cell Longev. 2022, 1300387. 10.1155/2022/1300387 35571235 PMC9095355

[B90] Wen J. He T. Qi F. Chen H. (2019). MiR-206-3p alleviates chronic constriction injury-induced neuropathic pain through targeting HDAC4. Exp. Anim. 68 (2), 213–220. 10.1538/expanim.18-0091 30587671 PMC6511522

[B91] Wu Y. Eisel U. L. M. (2023). Microglia-astrocyte communication in Alzheimer's disease. J. Alzheimers Dis. 95 (3), 785–803. 10.3233/JAD-230199 37638434 PMC10578295

[B92] Wu Y. Hu Q. Wu X. Cai Y. N. Zhang Y. Z. Wu Y. X. (2023). P7C3-A20 attenuates microglial inflammation and brain injury after ICH through activating the NAD(+)/Sirt3 pathway. Oxid. Med. Cell Longev. 2023, 7857760. 10.1155/2023/7857760 36819779 PMC9936507

[B93] Xia D. Y. Yuan J. L. Jiang X. C. Qi M. Lai N. S. Wu L. Y. (2021). SIRT1 promotes M2 microglia polarization via reducing ROS-mediated NLRP3 inflammasome signaling after subarachnoid hemorrhage. Front. Immunol. 12, 770744. 10.3389/fimmu.2021.770744 34899720 PMC8653696

[B94] Yang H. Ni W. Wei P. Li S. Gao X. Su J. (2021). HDAC inhibition reduces white matter injury after intracerebral hemorrhage. J. Cereb. Blood Flow. Metab. 41 (5), 958–974. 10.1177/0271678X20942613 32703113 PMC8054714

[B95] Zhang F. Qi L. Feng Q. Zhang B. Li X. Liu C. (2021). HIPK2 phosphorylates HDAC3 for NF-κB acetylation to ameliorate colitis-associated colorectal carcinoma and sepsis. Proc. Natl. Acad. Sci. U. S. A. 118 (28), e2021798118. 10.1073/pnas.2021798118 34244427 PMC8285910

[B96] Zhang L. Y. Zhang S. Y. Wen R. Zhang T. N. Yang N. (2024a). Role of histone deacetylases and their inhibitors in neurological diseases. Pharmacol. Res. 208, 107410. 10.1016/j.phrs.2024.107410 39276955

[B97] Zhang M. J. Zhao Q. C. Xia M. X. Chen J. Chen Y. T. Cao X. (2020). The HDAC3 inhibitor RGFP966 ameliorated ischemic brain damage by downregulating the AIM2 inflammasome. FASEB J. 34 (1), 648–662. 10.1096/fj.201900394RRR 31914678

[B98] Zhang S. Fujita Y. Matsuzaki R. Yamashita T. (2018). Class I histone deacetylase (HDAC) inhibitor CI-994 promotes functional recovery following spinal cord injury. Cell Death Dis. 9 (5), 460. 10.1038/s41419-018-0543-8 29700327 PMC5919919

[B99] Zhang Y. Zhao Y. F. Wang Y. N. Li J. Y. Huang Y. C. Lyu F. (2024b). Microglial histone deacetylase 2 is dispensable for functional and histological outcomes in a mouse model of traumatic brain injury. J. Cereb. Blood Flow. Metab. 44 (5), 817–835. 10.1177/0271678X231197173 38069842 PMC11197137

[B100] Zhang Z. Fang J. Zhou J. Ding F. Zhou G. Zhao X. (2022). Pterostilbene attenuates subarachnoid hemorrhage-induced brain injury through the SIRT1-dependent Nrf2 signaling pathway. Oxid. Med. Cell Longev. 2022, 3550204. 10.1155/2022/3550204 36506933 PMC9729048

[B101] Zhao H. Alam A. Chen Q. A Eusman M. Pal A. Eguchi S. (2017). The role of microglia in the pathobiology of neuropathic pain development: what do we know? Br. J. Anaesth. 118 (4), 504–516. 10.1093/bja/aex006 28403399

[B102] Zhao N. Li Y. Wang C. Xue Y. Peng L. Wang T. (2022b). DJ-1 activates the Atg5-Atg12-Atg16L1 complex via Sirt1 to influence microglial polarization and alleviate cerebral ischemia/reperfusion-induced inflammatory injury. Neurochem. Int. 157, 105341. 10.1016/j.neuint.2022.105341 35429577

[B103] Zhao Y. Mu H. Huang Y. Li S. Wang Y. Stetler R. A. (2022a). Microglia-specific deletion of histone deacetylase 3 promotes inflammation resolution, white matter integrity, and functional recovery in a mouse model of traumatic brain injury. J. Neuroinflammation 19 (1), 201. 10.1186/s12974-022-02563-2 35933343 PMC9357327

[B104] Zhou D. Jiang Y. (2019). Sirtuin 3 attenuates neuroinflammation-induced apoptosis in BV-2 microglia. Aging (Albany NY) 11 (20), 9075–9089. 10.18632/aging.102375 31631063 PMC6834423

[B105] Zhu H. Guan A. Liu J. Peng L. Zhang Z. Wang S. (2023). Noteworthy perspectives on microglia in neuropsychiatric disorders. J. Neuroinflammation 20 (1), 223. 10.1186/s12974-023-02901-y 37794488 PMC10548593

[B106] Zhu H. Guo Y. Huang A. Shen H. Chen Y. Song J. (2022). HDAC3-Regulated PGE2 production by microglia induces phobic anxiety susceptibility after stroke and pointedly exploiting a signal-targeted gamma visual stimulation new therapy. Front. Immunol. 13, 845678. 10.3389/fimmu.2022.845678 35251047 PMC8895955

[B107] Zhu W. Ma Z. (2023). SIRT2 overexpression decreases remifentanil-stimulated post-surgical hyperalgesia via microglia. Cell Mol. Biol. (Noisy-le-grand) 69 (12), 268–274. 10.14715/cmb/2023.69.12.42 38063126

